# Bacterial community composition and function along spatiotemporal connectivity gradients in the Danube floodplain (Vienna, Austria)

**DOI:** 10.1007/s00027-020-0700-x

**Published:** 2020-02-18

**Authors:** Magdalena J. Mayr, Katharina Besemer, Anna Sieczko, Katalin Demeter, Peter Peduzzi

**Affiliations:** 1 Department of Limnology and Oceanography, University of Vienna, Althanstrasse 14, 1090 Wien, Austria; 2 WasserCluster Lunz, Dr. Carl Kupelwieser Promenade 5, 3293 Lunz Am See, Austria; 3 Department of Thematic Studies–Environmental Change, Linköping University, Tema M, Campus Valla, 581 83 Linköping, Sweden

**Keywords:** Flooding, Hydrology, Dissolved organic matter, Freshwater bacterial diversity, Ecosystem processes

## Abstract

It is well recognized that river-floodplain systems contribute significantly to riverine ecosystem metabolism, and that bacteria are key players in the aquatic organic carbon cycle, but surprisingly few studies have linked bacterial community composition (BCC), function and carbon quality in these hydrologically highly dynamic habitats. We investigated aquatic BCC and extracellular enzymatic activity (EEA) related to dissolved organic carbon quality and algae composition, including the impact of a major flood event in one of the last remaining European semi-natural floodplain-systems. We found that surface connectivity of floodplain pools homogenizes BCC and EEA, whereas low connectivity led to increased BCC and EEA heterogeneity, supported by their relationship to electrical conductivity, an excellent indicator for surface connection strength. Hydrogeochemical parameters best explained variation of both BCC and EEA, while the algal community and chromophoric DOM properties explained only minor fractions of BCC variation. We conclude that intermittent surface connectivity and especially permanent isolation of floodplain pools from the main river channel may severely alter BCC and EEA, with potential consequences for nutrient cycling, ecological services and greenhouse gas emissions. Disentangling microbial structure–function coupling is therefore crucial, if we are to understand and predict the consequences of human alterations on these dynamic systems.

**Electronic supplementary material:**

The online version of this article (10.1007/s00027-020-0700-x) contains supplementary material, which is available to authorized users.

## Introduction

River-floodplain systems contribute significantly to riverine ecosystem metabolism (Battin et al. 2008). The prominent fluvial dynamics shaping river-floodplains, e.g. flow and flood pulses, create a temporal and spatial mosaic of habitats with differing hydrological conditions (Junk et al. 1989; Tockner et al. 2000), ranging from mainly groundwater-fed pools to fast flowing side arms. The hydrological regime affects physicochemical parameters, nutrient supply and the in- and outflux of microbes, organic and inorganic matter in the river-floodplain system (Tockner et al. 1999). Along the resulting strong environmental gradients heterotrophic bacteria transform or mineralize organic matter from various sources, thereby contributing to riverine carbon outgassing and sequestration (Luef et al. 2007; Peduzzi et al. 2008; Sieczko and Peduzzi 2014; Sieczko et al. 2015). Given the pivotal role of bacteria for the organic carbon cycle in aquatic ecosystems (Cole et al. 1988) and the importance of river-floodplain systems for riverine systems (Battin et al. 2008), surprisingly few studies have tried to link bacterial community composition and function to carbon quality and hydrological regime in river-floodplain systems (Besemer et al. 2005; Sieczko and Peduzzi 2014; Freimann et al. 2015).

Both, local and regional factors regulate the bacterial community composition in freshwater systems (Lindström and Langenheder 2012). Many environmental factors have been shown to act on the bacterial community composition, e.g. water temperature, nutrient concentration, pH and oxygen concentration (Besemer et al. 2005; Lindström et al. 2005; Shade et al. 2008). Dispersal might be an important factor during a flood pulse, but because environmental conditions change simultaneously, disentangling regional and local factors is difficult under environmental settings (Lindström and Langenheder 2012).

Terrestrially-derived organic carbon influx from the catchment and microbially-derived carbon (i.e. allochthonous and autochthounous sources, respectively) represent the primary resources for heterotrophic bacterial communities in river-floodplain systems (Besemer et al. 2009). When floodplain pools are disconnected from the main river channel, autochthonous dissolved organic matter (DOM), especially from algae, becomes more important than allochthonous inputs (Preiner et al. 2007; Besemer et al. 2009; Sieczko et al. 2015). Algal abundance and, notably, taxonomic affiliation, have been shown to impact bacterial communities, e.g., via their release of different organic metabolites (Eigemann et al. 2013; Bagatini et al. 2014), however their importance for bacterial community composition and function in floodplains remains unknown. Furthermore, bacterial utilization of allochthonous, aromatic material, which is introduced into the floodplains mainly during floods has been shown to support bacterial growth as indicated by extracellular enzymatic activity (Sieczko and Peduzzi 2014).

Bacteria use extracellular enzymes to degrade complex organic materials into assimilable molecules and enzymes are produced in response to available organic matter (Arnosti et al. 2014; Sieczko and Peduzzi 2014). Hence, extracellular enzymatic activity (EEA) has been used as a proxy for bacterial function with the aim to link bacterial communities to ecosystem functions under different environmental conditions (Peter et al. 2011; Lear et al. 2014).

The quality of dissolved organic carbon (DOC) can be an important driver of bacterial community composition (BCC) (Kirchman et al. 2004; Kritzberg et al. 2006) and of extracellular enzymatic activity (EEA) (Sieczko and Peduzzi 2014). Bacterial community composition and the suite of produced extracellular enzymes in turn influence DOC processing. Microbial diversity and community structure have been shown to influence the diversity and composition of the DOC compounds degraded, and the temporal patterns of DOC degradation (Singer et al. 2010; Logue et al. 2015). However, the structure–function relationship especially of microbial communities can be complex and is modified by functional redundancy and metabolic plasticity of the community (Allison and Martiny 2008). In an alpine floodplain, functional plasticity and redundancy were found to weaken the structure–function relationship of hyporheic bacterial communities in groundwater-fed and glacial streams (Freimann et al. 2013). Bacterial community composition and function may react differently to environmental changes and at other time scales in a way that the function may change faster than the bacterial community (Berga et al. 2012; Bier et al. 2015). This calls for simultaneous monitoring of both community structure and function to assess the influence of environmental change on microbial communities and organic matter processing in river-floodplain systems.

We investigated the bacterial community composition (BCC) and function (EEA) in six floodplain pools within the Donau-Auen National Park (Austria). These pools ranged from frequently connected to completely isolated from the main river channel, across 11 sampling dates from April to August. The aim of our study was to elucidate the relative importance of (1) hydrological parameters and water chemistry (hydrogeochemistry), (2) dissolved organic matter (DOM) quality and (3) algal community on structure (BCC) and function (EEA) of microbial communities during spring and summer in a year with an exceptionally strong flood. We tested the strength of the relationship between BCC and EEA during this period. Further, we attempted to elucidate the BCC and EEA in the floodplain pools during a historic 200-year flood event.

## Material and methods

### Study area and sampling procedure

The Donau-Auen National Park includes the free flowing stretch between Vienna and Bratislava and represents the last remaining semi-natural floodplain of the Upper Danube (Tockner and Stanford 2002). The floodplain pools consist of former river channels, which were decoupled from the flow regime in the nineteenth and twentieth century (Hohensinner et al. 2008). Hydrological connectivity of the floodplain water bodies has been increased through river restoration measures in the 1990s (Tockner and Schiemer 1997; Schiemer et al. 1999).

**Fig. 1:**
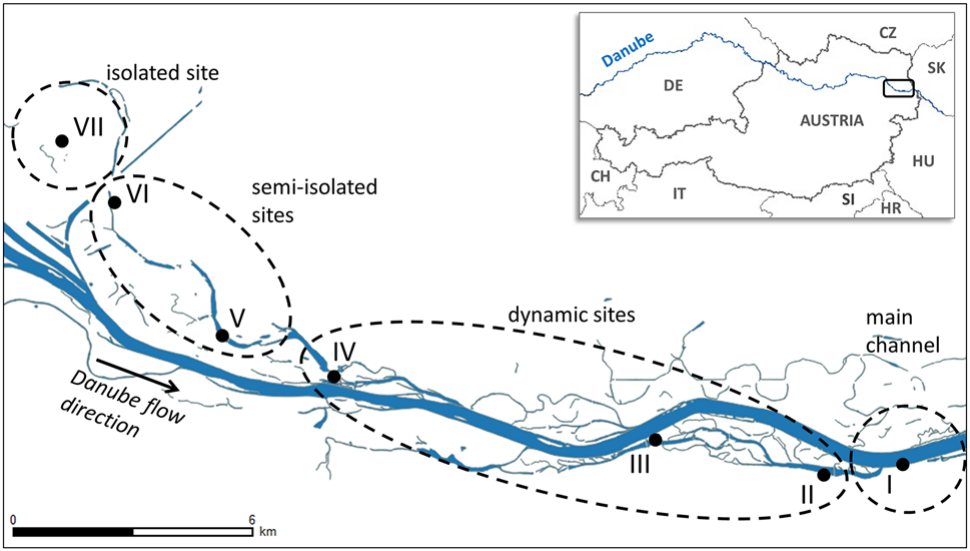
Map of the Danube floodplain showing the location of our sampling sites: main channel (I), dynamic sites (II–IV), semi-isolated sites (V, VI) and the isolated site (VII)

**Fig. 2:**
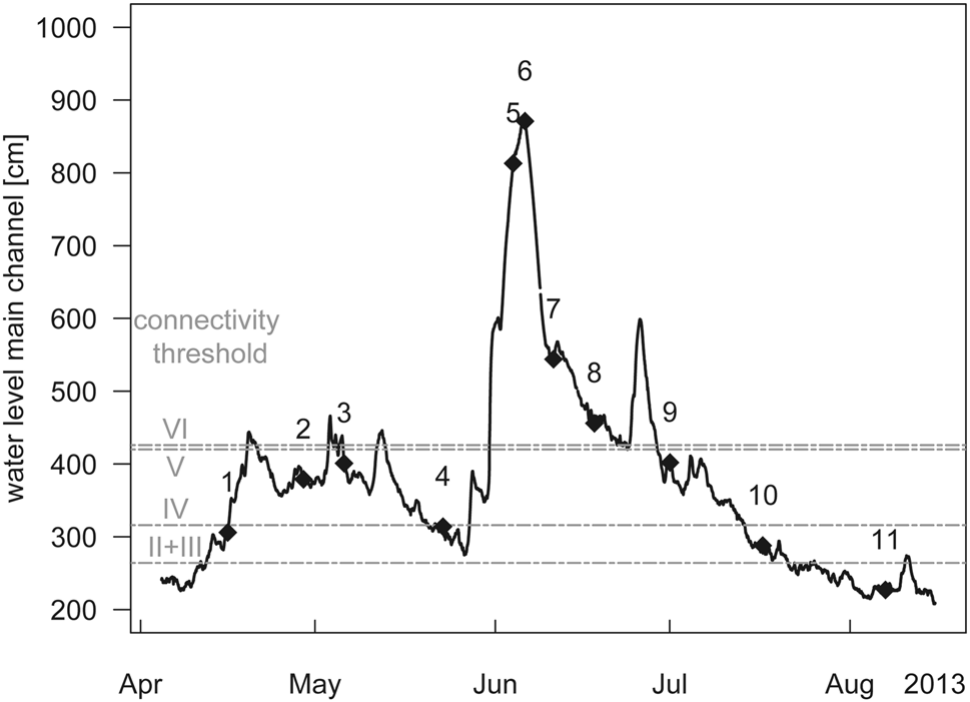
Hydrograph of the Danube main channel (I) at Wildungsmauer. Diamonds display sampling dates, which are numbered chronologically. Dashed lines indicate water levels at which individual floodplain pools get connected to the Danube main channel. The isolated site (VII) is never connected to the main channel

The main channel was sampled as a reference point at Wildungsmauer (I, Fig. 1). Dynamic sites (II, III, IV) are frequently connected to the main channel by surface flow on more than 180 days year^−1^ (Fig. 2). The semi-isolated sites V and VI are connected to the main channel at the downstream end of the floodplain pools under flooding conditions (station V 20–180 days year^−1^ and station VI 0–20 days year^−1^). The isolated site (VII) is located in a more elevated area without surface connection to the main channel. The latter sites are likely influenced by water level fluctuations below bankfull through groundwater seepage (Hohensinner et al. 2008). Samples were taken at 0.5 m depth on 11 dates in 2013: 16 April, 29 April, 06 May, 23 May, 04 June, 06 June, 11 June, 18 June, 01 July, 17 July and 07 August (Fig. 2), which were numbered chronologically (1–11) for easy identification in Fig. 4. Within this sampling period fell an exceptionally strong flood (samplings June 04 and 06 2013), which occurs, on average, once in 200 years (www.noel.gv.at). During the 200-year flood (04 and 06 Jun 2013), sites I, IV and V were not accessible. Samples from the main channel were instead taken about 37 km upstream within the district of the city Vienna, resulting in a total of 73 samples for further analysis.

### Hydrogeochemistry and hydrology

Water temperature, oxygen concentration, pH and electrical conductivity (WTW, Weilheim, Germany) were measured in situ. Standard methods were used to measure nitrate (DIN 38405-29), ammonium (DIN 38406-5) and soluble reactive phosphorus (SRP, EN ISO 6878). The organic content [%] of suspended solids (dry weight) was calculated as the ratio of ash-free dry weight to total dry weight. Water level records were provided by the government of Lower Austria, Department for Hydrology and Geoinformation.

### Bacterial community analysis (T-RFLP)

To track spatio-temporal patterns in the free-living bacteria (0.22–3 µm), we applied terminal restriction fragment length polymorphism (T-RFLP) profiling based on bacterial 16S rRNA genes. Filters were stored at − 80 °C until further processing. Filters were cut into pieces and DNA was extracted with the PowerSoil DNA Isolation Kit (MoBio, Carlsbad, CA, USA). Detailed PCR conditions are provided in the supplementary methods. PCR products were digested with the enzymes HhaI or HinfI and DNA fragments were detected with an ABI 3130 XL capillary sequencer (Applied Biosystems, Carlsbad, CA, USA). Relative contribution of an OTU to the community was calculated as the respective peak height divided by the cumulative peak height of a sample, peaks contributing less than 0.4% to a sample were removed. Both restriction enzymes were processed together. T-RFLP captures only the most abundant OTUs and as opposed to high-throughput sequencing methods taxonomic affiliation of OTUs is not provided. Nevertheless, T-RFLP has been shown to be highly reproducible and captures similar spatio-temporal patterns of the bacterial community composition (Besemer et al. 2012; Lindström et al. 2018).

For epifluorescence microscopy of prokaryotes and virus like particle abundance the protocol of Noble and Fuhrman (1998) modified after Chen et al. (2001) was used. Details are provided in the supplementary methods.

### Extracellular enzymatic activity

Six fluorogenic model substrates were used to determine potential extracellular enzymatic activities (Sieczko et al. 2015). 4-methylumbelliferyl (MUF) -α-d-glucoside, MUF-β-d-glucoside, MUF-β-d-xyloside, MUF-cellobioside and MUF-q-guanadinobenzoate were used as substrates to measure the enzymes α-glucosidase, β‐glucosidase, β-d-xylosidase, cellobiohydrolase and endopeptidase, respectively. For each sample a standard curve (0, 0.0033, 0.0331, 0.0662 µM MUF, Sigma) was established. Triplicates of each sample (3 ml, prefiltered 3 µm pore size) with model substrates at a final concentration of 3.02 µM were incubated for 12–156 min at in situ temperature and measured with a spectrofluorophotometer (RF-5301 PC Shimadzu, Kyoto, Japan) at an excitation 360 nm and emission 444 nm before and after the incubation. Extracellular enzymatic activities were calculated as µmol substrate hydrolyzed L^−1^ h^−1^ (Hoppe 1993). l-3.4-dihydroxyphenylalanine (L-DOPA) served as a substrate for measuring phenol oxidase activity (Pind et al. 1994). Prefiltered samples in triplicates (3 ml, 3 µm pore size) and a blank (MilliQ, Millipore) were mixed 1:1 with L-DOPA solution (5 mM in 2.5 mM NaHCO_3_ buffer, pH 8.3). The absorbance was measured with a spectrophotometer immediately at 460 nm (Hitachi U-2000, Tokyo, Japan) and after dark incubation for 82–173 min at in situ temperature. The phenol oxidase activity [µmol product L^−1^ h^−1^] was calculated using the molar absorption coefficient of 3700 for the L-DOPA product 3-dihydroindole-5.6-quinone-2-carboxylate (Mason 1948).

### Dissolved organic matter characteristics

Immediatelly after transportation to laboratory, water samples were filtered through combusted GF/F (500 °C, 0.7 µm pore size). The filtrate was stored in 4 °C in the dark in combusted (500 °C), acid washed glass vials until further analysis (up to 7 days) and used to assess quantity and quality of DOM. To obtain quantitative information on DOM, dissolved organic carbon concentration [mg l^-1^] (DOC) was measured with a TOC analyzer (Sievers 5310C, GE Analytical Instruments, Boulder, CO, USA). To aquire information on DOM quality, absorbance and fluorescence were measured simultaneously using an Aqualog spectrofluorometer (Horiba Scientific, Kyoto, Japan). From the corrected absorbance scans and excitation-emission matrices (EEM) indices describing quality of CDOM and FDOM were calculated. They included slope ratio (Sr) (Helms et al. 2008), carbon specific UV absorbance (SUVA_254_) (Weishaar et al. 2003), humification index (HIX) (Ohno 2002), freshness index (β:α) (Parlanti et al. 2000) and fluorescence index (FI) (McKnight et al. 2001). Details on the measurement procedure and calculation of indices are provided in the supplementary methods.

Individual components were modeled with Matlab (7.11.0) using the DOMFluor Toolbox (1.7; containing the N-Way toolbox, 3.1) by applying parallel factor analysis (PARAFAC) (Stedmon and Bro 2008; Andersson and Bro 2000). PARAFAC revealed two humic-like components (C1, C2) and one protein-like component (C3). Component 1 (Ex < 260 nm/Em 448–480 nm) and C2 (Ex 320–360 nm/Em 420–460 nm) correspond to high molecular weight (HMW), humic, terrestrial peaks A and C whereas Component 3 (Ex 270–280 (240)/Em 330–368) resembles tryptophan-like peak T (Fellman et al. 2010).

### HPLC and CHEMTAX analysis of phytoplankton pigments

The phytoplankton pigments were analysed with HLPC (VWR Hitachi Elite LaChrom) and pigment identification was based on retention time and absorption spectra. Algal classes were estimated from chlorophyll and carotenoid pigments with CHEMTAX (Mackey et al. 1996; Wright et al. 1991) using the version 1.95. The program determines algal class abundances based on estimates of pigment ratios. Details on the HPLC and CHEMTAX analysis are provided in the supplementary methods.

### Data analysis

Multivariate statistics and graphs were performed in R 2.15.1 using the packages vegan, MASS, packfor, nlme and car (Venables and Ripley 2002; Blanchet et al. 2008; Fox and Weisberg 2011; Pinheiro et al. 2011; Oksanen et al. 2013). BCC and EEA patterns were visualized using NMDS plots based on Bray–Curtis dissimilarities of T-RFLP data and Euclidean distances of square root transformed, *z*-score standardized extracellular enzymatic activities. To test for differences between site categories in BCC and EEA, an analysis of similarities (ANOSIM) was used (999 permutations and *p* values were Bonferroni corrected).

A forward selection procedure according to Blanchet et al. (2008) was performed (9999 permutations) to select a subset of CDOM quality, algal classes and environment explaining significant parts of Hellinger-transformed BCC and EEA (transformed as above). Explanatory variables were *z*-score standardized prior to all analyses. Explanatory variables with variance inflation factors above 10 were removed sequentially. RDA-based variation partitioning (Peres-Neto et al. 2006) was used to partition the explained BCC and EEA variation between CDOM quality, algal classes and environment. Mantel statistic with Spearman’s rank correlation was performed to investigate the relationship between BCC (Bray–Curtis dissimilarities of T-RFLP data) and EEA (square root transformed, *z*-score standardized extracellular enzymatic activities). A partial Mantel statistic was done to control for the effect of the environment. Environmental variables were selected by a forward selection procedure for BCC and EEA, respectively, using the previously selected variables from cDOM quality, algal classes and hydrogeochemistry. We combined the explanatory variables of BCC and EEA and removed those with variance inflation factors above 10. The obtained explanatory variables were used as the third matrix in the partial Mantel statistic.

## Results

### Hydrological and physicochemical conditions

The hydrological conditions in the Danube varied considerably during our sampling campaign from April to August 2013. A historical flood at the beginning of June caused running water conditions in the dynamic and semi-isolated sites in the Danube floodplain (sampling numbers 5–7; Fig. 2). Further, small variations in water level led to different extent of connectivity between the main channel and the floodplain pools, which become connected to the main channel at a certain water level (Fig. 2). Over the sampling period water temperatures ranged from 9.2 to 23 °C in the main channel, 10.7–31.4 °C at the dynamic sites, 11.9–27.7 °C at the semi-isolated sites and 11.1–20.7 °C at the isolated site. Electrical conductivity fluctuated between 280 and 474 µS cm^−1^ in the main channel, 312 and 525 µS cm^−1^ at the dynamic sites, 330 and 664 µS cm^−1^ at the semi-isolated sites and 560 and 1600 µS cm^−1^ at the isolated site. For a summary of site characteristics see Supplementary Table 1.

### BCC and EEA along the connectivity gradient

**Fig. 3:**
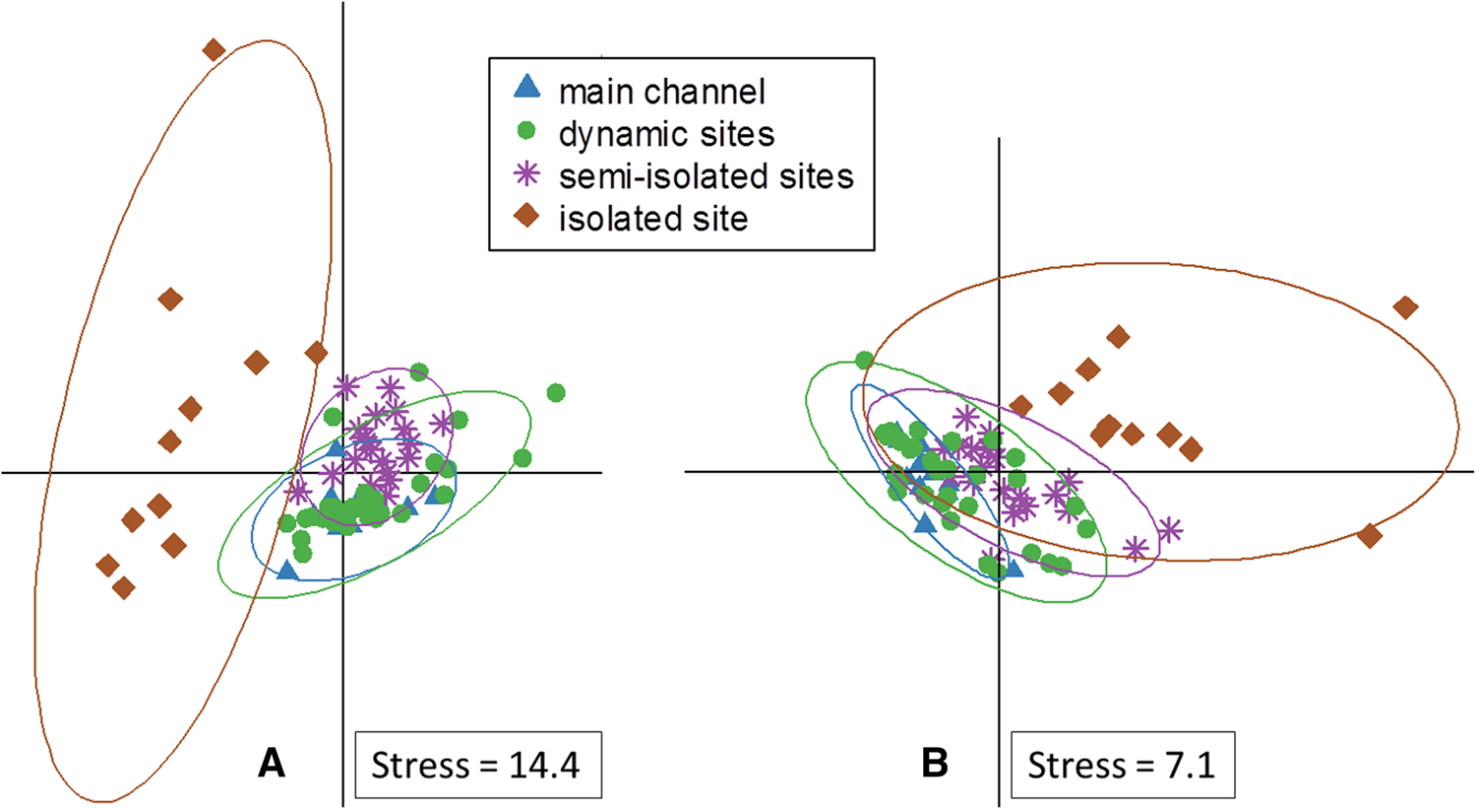
Non-metric multidimensional scaling plots covering the whole sampling period of **a** bacterioplankton community compositions (BCC) based on Bray–Curtis dissimilarities of T-RFLP data and **b** extracellular enzymatic activity (EEA) based on a euclidean distance matrix

Patterns of the bacterioplankton community composition, analyzed by T-RFLP (Moeseneder et al. 1999), revealed a total of 91 and 84 OTUs with the restriction enzymes HinfI and HhaI, respectively. On average 29 OTUs (same for HinfI and HhaI) were found per sample. Considering the whole sampling period, which included a 200-year flood, the BCCs of the dynamic sites were statistically indistinguishable from the BCCs from the main channel, while the BCCs from the semi-isolated sites differed significantly from both, the main channel and the dynamic sites (Fig. 3a; ANOSIM, *p* < 0.01, Supplementary Table 2). BCCs of the isolated site were clearly distinct from all other sites (ANOSIM, *p* < 0.01) showing a pronounced temporal variability (Fig. 3a).

Analysis of microbial function was assessed via potential extracellular enzymatic activities (EEA), which showed that over the entire sampling period main channel and dynamic sites overlapped (Fig. 3b; ANSOIM, *p* > 0.05, Supplementary Table 2). EEA at the main channel differed significantly (ANOSIM, *p* < 0.05, Supplementary Table 2) from EEA at the semi-isolated sites, though their patterns overlapped (Fig. 3b). Similar to the BCC, the isolated site showed a distinct EEA (ANOSIM *p* < 0.01) with a high temporal variation (Fig. 3a, b).

A mantel statistic showed that BCC and EEA were significantly correlated (Mantel test, Pearson, *R* = 0.61, *p* = 0.001). A partial mantel statistic was performed to see direct effects of the BCC on the EEA (omitting the isolated site, because of its distinct character), while partialling out the environment. To perform the analysis, a set of environmental variables was selected by a forward selection procedure. A weak partial correlation was found between BCC and EEA with a Mantel statistic *R* = 0.20 and *p* < 0.01 (Pearson).

### Influence of temporal water level changes on BCC and EEA

**Fig. 4:**
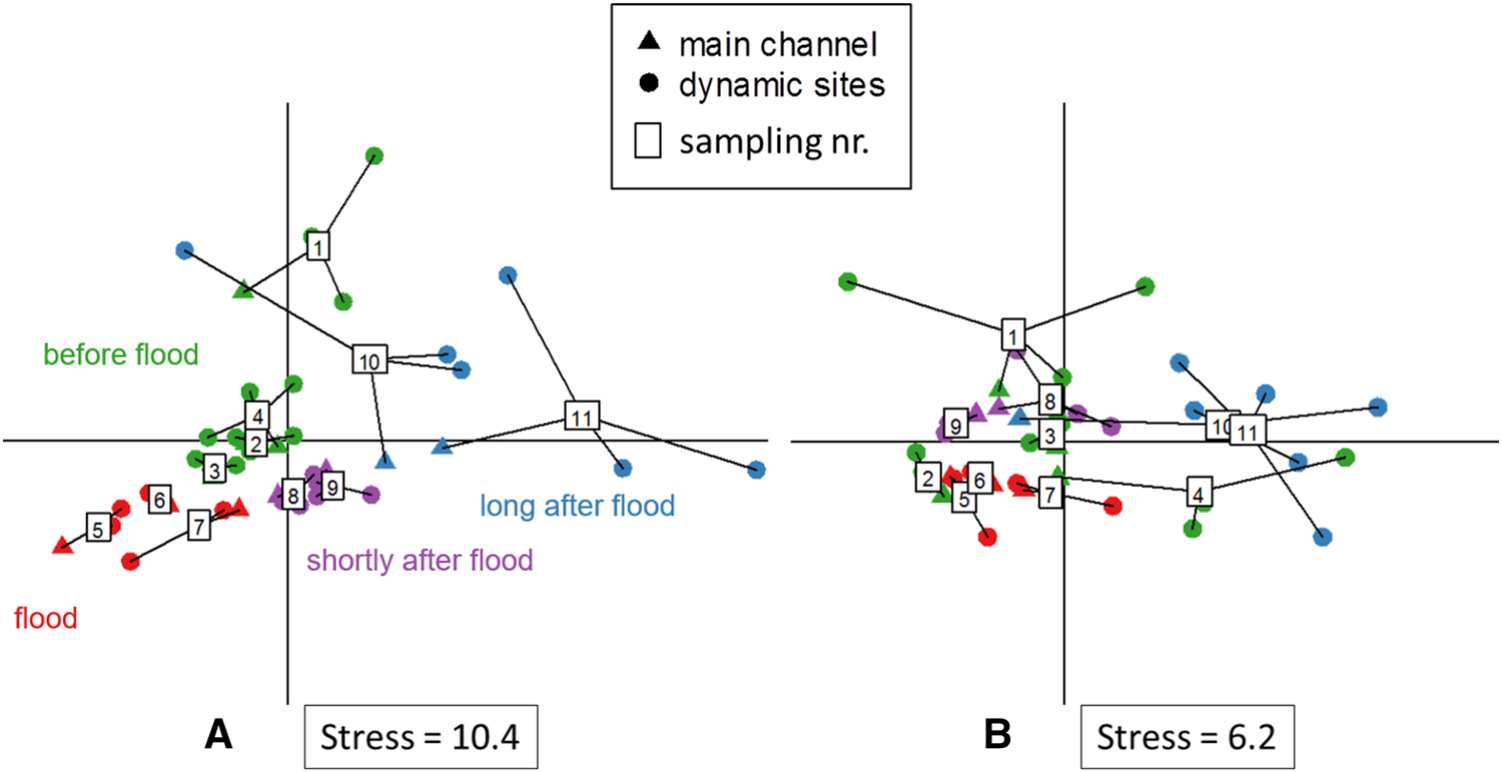
Non-metric multidimensional scaling plots of **a** BCC and **b** EEA in the main channel and at the three dynamic sites (II–IV). Boxes represent the group centroid of the samples at each sampling date and the numbers (1–11) in boxes identify the sampling dates (see Fig. 2). Hydrological phases: before flood (sampling number: 1–4), flood (5–7), short after flood (8–9) and long after flood (10–11)

To assess the influence of temporal changes in connectivity on microbial structure and function, we focused on the relations between the main channel and dynamic sites. NMDS was used to visualize changes in the BCC and EEA at these sites at a finer resolution (Fig. 4). The main channel and dynamic sites-BCCs showed a clear temporal pattern with clustering of hydrological phases (Fig. 4a). Furthermore, at water levels well above mean water, indicating a strong surface connection between main channel and dynamic sites, the BCCs within each sampling date were very similar to each other (Fig. 4a). In contrast, under conditions close to mean water level (293 cm) and below, the BCCs diverged from each other (sampling dates 1, 10, 11). The only exception was date 4 (Fig. 4a), which was characterized by mean water level following a period of elevated water levels (Fig. 2); samples from this date showed low variation between sampling sites and clustered with the samples from the preceding sampling dates. The EEA showed less pronounced temporal patterns (Fig. 4b) than the BCC. Still, the variability among sampling dates was lower at strong surface connectivity and higher when sites were less connected (Fig. 4b), similarly to the BCC (Fig. 4a). Notably, EEA of samples from date 4 exhibited strong variation between sites and were more similar to other low-water level samples, contrasting the patterns observed for BCCs (Fig. 4a, b).

**Fig. 5:**
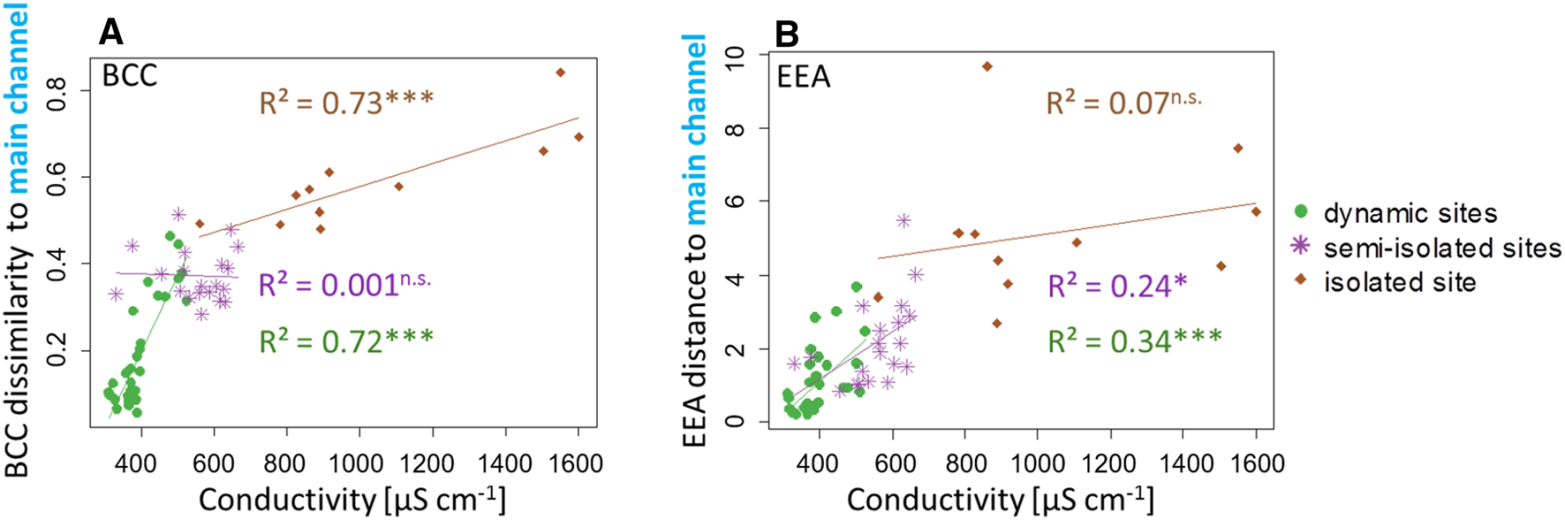
**a** Bray–Curtis dissimilarities of bacterioplankton community compositions (BCC), based on T-RFLP between main channel and respective sites within each sampling date were plotted against conductivity of the respective site. **b** Euclidean distances of extracellular enzymatic activities (EEA, square root transformed, centered and scaled) between main channel and respective sites within each sampling date were plotted against conductivity of the respective site. Linear regressions were performed within site categories. Stars indicate significance levels: *** < 0.001, ** < 0.01, * < 0.05, n.s. > 0.05

To further explore the water level-related patterns in BCC and EEA, the Bray–Curtis dissimilarity of BCC and the Eucledian distance of EEA between main channel and the respective sites at each sampling date were plotted against the electrical conductivity, which is strongly related to hydrology in this floodplain (Weigelhofer et al. 2015). The relationship between conductivity and BCC dissimilarity to the main channel was significant for the dynamic sites and the isolated site, but not for the semi-isolated sites (Fig. 5a). However, considering all sites, a significant relationship between conductivity and BCC dissimilarity (linear regression, *R*^2^ = 0.68, *p* < 0.001) was found. Conductivity and EEA distance to main channel were significantly correlated at dynamic sites and semi-isolated sites, but no relationship was found at the isolated site (Fig. 5b). With respect to all sites a clear conductivity—EEA distance correlation was found (linear regression, *R*^2^ = 0.57, *p* < 0.001).

### Potential drivers of BCC and EEA

**Fig. 6:**
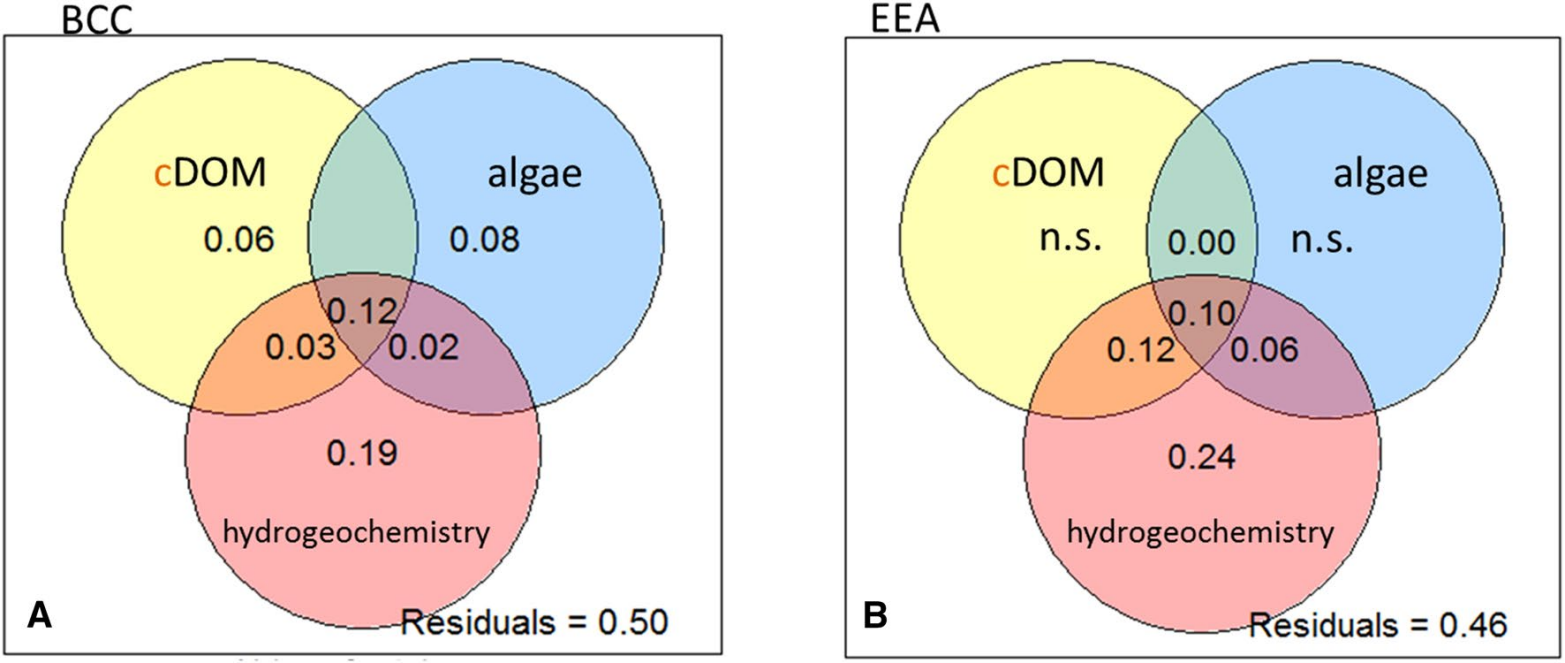
Venn diagramm of **a** BCC and **b** EEA visualizing the respective explained variance by three groups of variables, cDOM quality (cDOM), algae classes (algae), hydrogeochemical parameters (hydrogeochemistry). Variables were selected by forward selection and are listed in Table 1

Three groups of variables, namely cDOM quality, algal classes and hydrogeochemistry, compiled by a forward selection procedure (Table 1), were tested for their respective contribution to explain BCC and EEA variation. The isolated site was omitted from this analysis, because of its unique BCC and EEA, and its permanent disconnection from the main channel. This leads to a different site character, because this pool never experiences surface exchange of nutrients, organic matter or organisms even during exceptionally high water level in the main channel. Variation partitioning showed that in total 50% of the BCC variation could be explained by these three groups of variables (Fig. 6a). The group hydrogeochemistry contributed most to the explained variation. cDOM properties and algae showed small significant individual contributions to the overall explained variation (6% and 8%, respectively; Fig. 6a). The variation in the EEA could be explained to a total extent of 54% (Fig. 6b). While hydrogeochemistry explained the highest individual fraction (24%), pure effects of cDOM and algae did not significantly explain variation in the EEA.

**Table 1:** Results of a forward selection based on an RDA and 9999 permutations, the three groups (chromophoric dissolved organic matter (CDOM) quality, algal classes, hydrogeochemistry) were analyzed separately

*n* = 61	Results of forward selection applied to main channel, dynamic sites and semi-isolated sites	Adjusted *R*^2^ (*p* < 0.05)
Bacterioplankton community composition		
CDOM quality	Aromaticity (SUVA), fluorescence index (FI), freshness index (β:α), humic-like A, molecular weight (SR), humification (HIX)	Adj. *R*^2^ = 0.20
Algal classes	Euglenophytes, pheophytin a, diatoms, cyanoprokaryotes, dinoflagellates, chlorophytes	Adj. *R*^2^ = 0.22
Hydrogeochemistry	Nitrate, water temperature, pH, water level main channel, phosphate, oxygen, water age, conductivity	Adj. *R*^2^ = 0.36
Extracellular enzymatic activity		
CDOM quality	Aromaticity (SUVA), freshness index (β:α)	Adj. *R*^2^ = 0.24
Algal classes	Cryptophytes	Adj. *R*^2^ = 0.16
Hydrogeochemistry	Water temperature, organic suspended solids%, oxygen	Adj. *R*^2^ = 0.52

Main channel, dynamic sites and semi-isolated site were included in the analysis

## Discussion

This study aimed at gaining new insights into the effects of a major flood event on the composition and function of the aquatic bacterial community in one of the last remaining European semi-natural floodplain systems. Our results suggest that the hydrological connectivity and the associated hydrogeochemical dynamics strongly shape the BCC and EEA on spatial and temporal scales, whereas the algae community and cDOM properties were of lesser importance.

### Spatiotemporal patterns of BCC and EEA under different hydrological settings

High water levels of the Danube main channel exacerbated a strong, but temporary homogenizing effect on both BCC and EEA in the dynamic sites (Fig. 4). Considering the entire sampling period BCC and EEA in the dynamic sites were statistically indistinguishable from the main channel (Fig. 3). Notably, during our sampling period the dynamic sites were connected to the main channel most of the time, which probably explains the overall similarity of BCC and EEA through dispersal via transport of bacterioplankton with the main channel water, as well as homogenization of hydrogeochemical site conditions (Lindström and Langenheder 2012). However, once the floodplain pools lost their strong surface connection to the main channel, the BCC dissimilarity between main channel and the dynamic sites increased, which may indicate a shift from a regionally to a more locally determined BCC and function (Lindström and Langenheder 2012). Neutral effects or species sorting according to local environmental conditions could be driving the changes in BCC following disconnection (Lindström and Langenheder 2012; Bozelli et al. 2015). This raises the question of which local environmental conditions are responsible. Our findings agree with an earlier study on zooplankton communities in floodplain pools, which found that floods acted as erasers of environmental and ecological peculiarities created during phases of low connectivity (Hay et al. 2000a; Medley and Havel 2007; Lesack and Marsh 2010; Bozelli et al. 2015).

A closer look at the BCC in the main channel and the dynamic sites revealed a clear temporal pattern with hydrological phases before, during, shortly and long after the flood clustering together (Fig. 4a). The BCC shifted during the flood, but afterwards the community re-established similar to that before the flood event (Fig. 3a). The flood might supplement the BCC with bacteria from flooded soil, adjacent water bodies and, due to stronger current, detached bacteria from the river bed (Tekwani et al. 2013). Further, species sorting may act through altered environmental conditions, as, e.g., significant decrease of algae biomass during flooding conditions and drastical increase of inorganic particle load (Tockner et al. 1999; Limberger and Schagerl 2005).

In contrast to the BCC, EEA did not follow such a clear temporal pattern (Fig. 3b). One explanation could be that function can change faster than the composition in microbial communities (Berga et al. 2012; Bier et al. 2015). This could explain sampling date 4, when BCC was similar to that of other high connectivity-dates, whereas EEA already resembled that of disconnected phases (Fig. 3). While the BCC might bear a historical imprint of previously active bacterial populations (Andersson et al. 2014), this might not be true to the same extent for EEA. Nevertheless, EEAs during the flood clustered together, likely reflecting the harsh environmental conditions during this event.

### BCC and EEA in the permanently isolated floodplain pool

Despite the major flood event in our study period, BCC in the isolated floodplain pool differed clearly from the main channel and the periodically connected floodplain pools at all measured time points (Fig. 3), underlining the importance of surface connectivity for BCC and EEA. Groundwater inundation was apparently not sufficient to influence the microbial community through changes in hydrochemical conditions (Tockner et al. 2000). We found the highest temporal variability within the BCC in the isolated site, which is in agreement with results from an earlier study in the Danube river-floodplain system, which found pronounced seasonal patterns of bacterial communities in isolated floodplain pools (Besemer et al. 2005). Consistent with the BCC, EEA in the isolated site showed a distinct and more fluctuating pattern than observed in the other sites, suggesting that the BCC of the isolated site is not only taxonomically, but also functionally unique within this study area (Fig. 3b).

### Linking composition and function of the aquatic bacterial community

BCC and EEA were significantly correlated, but after correcting for environmental factors only a very weak correlation remained. This could be due to strong and independent environmental forcing of EEA, overruling the effect of BCC on EEA, or to simultaneous environmental forcing of EEA and BCC. Linking microbial community structure to function is still a challenge in microbial ecology research (Bier et al. 2015), due to the high functional redundancy and metabolic plasticity often found in bacterial communities (Allison and Martiny 2008; Comte et al. 2013; Wagner et al. 2014). Determining when and where BCC and EEA are linked might help us in the future to better predict ecosystem processes (Graham et al. 2016).

### Influence of hydrogeochemistry, algal classes and cDOM on BCC and EEA

Hydrogeochemical parameters were most important for explaining variation in BCC and EEA (Fig. 5; Table 1). The hydrogeochemical parameters chosen by forward selection included variables, which have been shown to influence bacterial community structure and function before, namely nutrient concentration (Besemer et al. 2005; Winter et al. 2007), water temperature (Lindström et al. 2005), pH (Lindström et al. 2005; Fierer et al. 2007), oxygen concentration (Shade et al. 2008) and organic particle concentration (Wilczek et al. 2005). Also, hydrology-related parameters, like water level, water age and electrical conductivity were among the potential drivers of BCC, emphasizing the impact of hydrological connectivity on BCC (Besemer et al. 2005). Hydrology has also been found to be an important regulator of the BCC in an Amazonian floodplain lake and alpine floodplains (Freimann et al. 2015; Melo et al. 2019).

The weaker, but significant influence of the algal community on BCC agrees with earlier findings in other habitats (Eigemann et al. 2013; Bagatini et al. 2014) and suggests interactions between these taxonomic groups, such as bacteria tracking changes in algal-derived organic matter, for instance (Paver et al. 2013). However, this effect on microbial community structure was not reflected in community functioning. The apparently neglectable influence of the algal community on EEA contradicts our hypothesis and contrasts a previous study in the Danube river-floodplain system, which suggested a strong correlation of chlorophyll-a, primary productivity and EEA during a post-flood period (Sieczko et al. 2015). The relative importance of the algae community is likely higher at receding water levels and might be masked by the strong impact of the flood event on BCC and EEA in the present study.

Similarly, cDOM affected BCC, but not EEA. Differential environmental impact on structure and function is not unusual for microbial communities and is indicative for functional redundancy (Frossard et al. 2012). The weak correlation between BCC and EEA remaining after correcting for the influence of environmental variables also suggests a certain level of functional redundancy and/or metabolic plasticity in these communities (Allison and Martiny 2008; Comte et al. 2013). Further, cDOM is tightly linked to hydrological conditions in this floodplain (Besemer et al. 2009; Sieczko and Peduzzi 2014), so any potential relationship between cDOM and EEA could be masked by strong and simultaneous control by hydrology.

The limitations of fingerprinting methods, like T-RFLP, for assessing bacterial richness and community structure have been discussed in several previous studies. T-RFLP targets bacterial community patterns of only the most abundant OTUs, and the number of OTUs detected may depend on the rank-abundance distribution rather than on the actual richness of the community (Blackwood et al. 2007; Bent and Forney 2008; Besemer et al. 2012). Also, in contrast to high-throughput sequencing methods, T-RFLP does not provide any information on the taxonomic affiliation of the detected organisms. However, its high reproducibility and relatively low costs have rendered T-RFLP a highly popular and still widely used method for microbial community structure analysis (e.g. Lindström et al. 2018; Trivedi et al. 2019). Several studies comparing high-throughput sequencing methods and T-RFLP for the analysis of bacterial or fungal communities concluded that T-RFLP generates reliable general community patterns and is a good option if resources for high-throughput sequencing methods are limited (Lindström et al. 2018). Similarly, measuring the extracellular enzymatic activity captures only part of the microbial functioning, which has recently been shown to be more complex than previously thought in aquatic environments (Reintjes et al. 2017). Nevertheless, combining BCC and EEA analysis with measurements of cDOM, algae community and a range of hydrogeochemical parameters holds the potential to elucidate the bacterial community-function relationship and controlling factors, which are still under much debate (Bier et al. 2015; Graham et al. 2016).

## Conclusions

Generally, we found that both, smaller increases of surface connectivity and historic flood pulse had a homogenizing effect on the BCC and EEA of the dynamic sites within the floodplain. Disconnection of dynamic sites before as well as after the flood increased both, BCC and EEA dissimilarity between the sites, indicating a shift from a regionally to a locally determined BCC and EEA. Hydrological parameters and water chemistry (hydrogeochemistry) contributed most to the explained variance of BCC and EEA, which might be due to the exceptional flood event during our sampling period. Algal community and cDOM properties explained only a small fraction of the BCC and was not siginifcantly explaining additional variance of EEA, indicating that during a period with a strong flood event other factors potentially shaping BCC and EEA may be overruled. Most of the European floodplains, once bordering all rivers, are only remnants of their original area. Further studies revealing the taxonomic identity and associated functional potential of the floodplain BCC might yield further insights into consequences of hydrological connectivity and other factors on BCC and EEA. Remaining floodplains are under anthropogenic pressure and prone to isolation through river incision (Amoros and Bornette 2002), for instance. Our data suggest that especially permanent isolation of floodplain pools from the main river channel severely alters BCC and EEA, with potential consequences for nutrient cycling, ecological services and greenhouse gas emissions. Knowledge on microbial structure–function coupling is therefore crucial, if we are to understand and predict the consequences of human alterations on these dynamic systems.

## Electronic supplementary material

Below is the link to the electronic supplementary material.

Supplementary file1 (DOCX 35 kb)
