# Supplementary material for: Bacterial community composition and function along spatiotemporal connectivity gradients in the Danube floodplain (Vienna, Austria)
Source: Aquat Sci. 2020 Feb 18;82(2):28. doi: 10.1007/s00027-020-0700-x (PMC7045780; doi:10.1007/s00027-020-0700-x)
Supplement: Supplementary file 1 — Supplementary file1 (DOCX 35 kb) [file 27_2020_700_MOESM1_ESM.docx]

Bacterial community composition and function along spatiotemporal connectivity gradients in the Danube floodplain (Vienna, Austria)

Aquatic Sciences

Magdalena J. Mayr, Katharina Besemer, Anna Sieczko, Katalin Demeter, Peter Peduzzi

Supplementary Methods

**Bacterial community analysis (T-RFLP)**

To track spatio-temporal patterns in the BCC, we applied terminal restriction fragment length polymorphism (T-RFLP) proﬁling based on bacterial 16S rRNA genes. 100-400mL water were prefiltered with a 3µm TSTP pore size filter (Millipore, Billerica, MA, USA) to remove large particles, algae and grazers and then ﬁltered onto a 0.22µm ﬁlter (GSWP, Millipore) to target the free-living bacterial community (0.22 – 3µm) as opposed to the particle associated bacterial community (>3µm). Filters were stored at -80°C until the DNA was extracted with the PowerSoil DNA Isolation Kit (MoBio, Carlsbad, CA, USA). Filters were cut into pieces prior to extraction. The bacteria speciﬁc hexachloro-ﬂuorescein-labeled primer 27F-hex (5’-AGR GTT TGA TCM TGG CTC AG-3’) and the universal primer 519R (5’-GWA TTA CCG CGG CKG CTG-3’; Life Technologies, Carlsbad, CA, USA) were used at 0.4µmol L−1 in the PCR reactions. Each reaction contained 0.2mmol L−1 of each dNTP, 1.25U Taq Polymerase with recommended buﬀer, 50µg bovine serum albumin, 2mmol L−1 MgCl2 (Thermo Fisher Scientiﬁc, Waltham, MA, USA) and 3µL extracted DNA in a ﬁnal volume of 50µL. Samples without DNA served as a negative control. Samples were ampliﬁed by 3min initial denaturation at 94°C, 25 cycles with 45s denaturation at 94°C, 45s annealing at 55°C, 60s elongation at 72°C and 10min ﬁnal elongation at 72°C.

The PCR product was checked with agarose gel electrophoresis and puriﬁed with the QIAquick PCR Puriﬁcation Kit (Qiagen, Hilden, Germany). PCR products were digested for 16h at 37°C containing 10U of enzymes HhaI or HinfI (Thermo Fisher Scientiﬁc) and 200ng PCR product in a ﬁnal volume of 20µL. The samples were desalted with Sephadex G50 Superﬁne (Sigma-Alrdich, St. Louis, MO, USA) and denatured for 3min at 95°C with Hi-Di formamide and an internal size standard (MapMarker1000-ROX, BioVentures Inc, Murfreesboro, TN, USA). Labelled DNA fragments were detected with an ABI 3130 XL capillary sequencer (Applied Biosystems, Carlsbad, CA, USA) and processed with the Peak Scanner Software 1.0 (Applied Biosystems). Peak assignment, including fragment lengths of 50-492 base pairs, to operational taxonomic units (OTUs) was checked manually.

### HPLC analysis of phytoplankton pigments

50-1000ml of a sample were filtered onto GF/F filters (Whatman, GE Healthcare, Chicago, IL, USA) and stored at -80°C. To extract the pigments the filter was cut and 5ml 90% acetone glass beads (0.5mm diameter, Carl Roth, Karlsruhe Germany) were added. The filter was mashed with a FastPrep (FastPrep-24, MP Biomedicals, Irivine, CA, USA) for 1min at maximum speed and stored at 5°C for extraction for 8 – 16 hours. After centrifuging twice for 10 min at 4000rpm at 4°C (5810 R Eppendorf, Hamburg, Germany) the supernatant was subjected to HPLC analysis (VWR Hitachi Elite LaChrom) equipped with an autosampler (L-2200, 10°C), pump (L-2130), column oven (L-2300,35°C), C18-column with precolumn and a diode array detector (L-2455). The mobile phase consisted of methanol: 0.5M ammonium acetate (80:20), acetonitrile:water (90:10) and ethyl acetate (Wright et al. 1991). Pigment identification was based on retention time and absorption spectra. Pigment standards (DHI, Horsholm, Denmark, Sigma-Aldrich, Inochem-Frontier Scientific, Logan, UT, USA) were used for calibration.

**CHEMTAX analysis of phytoplankton pigments**

Algal classes were estimated from chlorophyll and carotenoid pigments with CHEMTAX (Mackey et al. 1996) using the version 1.95. The program determines algal class abundances based on estimates of pigment ratios. To gain homogeneous data sets, a cluster analysis of environmental parameters was done and the resulting six groups were processed separately in the CHEMTAX analysis. An initial pigment ratio matrix (Supplementary table 3) was created from literature values (Descy et al. 2000; Schagerl and Donabaum 2003; Schluter et al. 2006; Greisberger and Teubner 2007; Lauridsen et al. 2011). Sixty randomized pigment ratio matrices were derived from the initial ratio matrix multiplied by F = 1 + 0.7 * (R - 0.5), where R is a random number between 0 and 1. The mean of the best six CHEMTAX results was calculated and used for further analysis. Results are given as chlorophyll-a (chl-a) equivalents, so all groups sum up to the total chl-a concentration of a sample. Samples with pheophytin-a to chlorophyll-a ratios higher than 0.5 were removed from the analysis. Ratio limits were set to 500 for the pigment ratios and to 100 for chl-a (default).

### Epifluorescence microscopy of prokaryotes and virus like particle abundance

Unfiltered samples were fixed with formaldehyde to 2% final concentration. Subsamples of 0.1 – 0.5ml were filtered onto an Anodisc filter (Whatman, 0.02µm pore size) on top of a supporting filter (Millipore, 0.45µm pore size). The top filter was stained with a drop of SYBR gold (Invitrogen Carlsbad, CA, USA) with a concentration of 3.3x, mounted on a slide with Citifluor (AF1, Agar scientific, Stansted, UK) and stored frozen until analysis. Per sample 332 - 768 prokaryotic cells (mean 464) and 269 – 986 virus like particles (mean 478) were counted by epifluorescence microscopy.

### Dissolved organic matter characteristics

Measurements for chromophoric dissolved organic matter (CDOM) and fluorescent dissolved organic matter (FDOM) were performed simultanously using 1cm quartz cuvette with Aqualog spectrofluorometer (Horiba Scientific, Kyoto, Japan). For CDOM the absorbance scans were collected between 240 nm and 600 nm at 1nm intervals. For FDOM, the excitation-emission matrices (EEM) were collected with fluorescence excitation wavelengths every 1 nm (240-600nm) and emission wavelengths every 3 nm (214 – 620nm). All spectra were corrected for blank (MQ water). Fluorescence spectra were also Raman normalized and corrected for inner-filter effect (Stedmon et al., 2003, Fasching et al., 2014). To characterize CDOM several indices were calculated. They included: molecular weight estimation Slope ratio (Sr) (Helms et al. 2008) and carbon specific UV absorbance (SUVA254) (Weishaar et al. 2003). Sr was used to obtain information about changes in molecular weight of DOM and it was calculated as the ratio of two log-transformed absorption spectra (275-295 nm) and (350-400 nm), Sr is inversely correlated with molecular weight of DOM. SUVA254 [l mg-1 m-1] was used to obtain an aromaticity index of DOC. It was acquired as a ratio of absorption at 254 nm to the DOC concentration. SUVA254 is positively correlated with DOC aromaticity.

The indices used to describe FDOM, included freshness index (β:α) (Parlanti et al. 2000) and humification index (HIX) (Ohno 2002). β:α served as an indicator of recently produced DOM (autochthonous input), β : α was calculated as the ratio of emission intensity at 380 nm divided by the emission intensity maximum observed between 420 and 435 nm, obtained at excitation 310 nm. β represents more recently derived DOM and α represents more decomposed DOM. HIX served as indicator of humic substance content and it was calculated following Ohno (2002). HIX is positively correlated with the degree of humification of DOM Additionaly the fluorescence index (FI) was calculated by using filtered and acidified (HCl, pH 2) samples which were measured with a spectrofluorophotometer (RF-5301 PC, Shimadzu) using 1 cm quarz cuvette. The ratio of fluorescence emission intensity at 450 nm to 500 nm obtained under excitation of 370 nm was calculated. The lower values indicate more terrestrially derived DOM (allochthonous) whereas higher imply more microbially derived DOM (autochthonous) (McKnight et al. 2001).

Supplementary Tables

Supplementary table 1 Water chemistry and biological characteristics of the different floodplain sites

|  | Main channel | Dynamic sites | Semi-isolated sites | Isolated site |
| --- | --- | --- | --- | --- |
| Water temperature (°C) | 9.2-23 | 10.7-31.4 | 11.9-27.7 | 11.1-20.7 |
| Conductivity (µS cm^-1^) | 280-474 | 312-525 | 330-664 | 560-1600 |
| Nitrate(mg l^-1^) | 6.6-11.6 | 0-12.7 | 0-10.1 | 0-0.9 |
| Phosphate (µg l^-1^) | 0-33 | 1-31 | 0-8 | 7-397 |
| pH | 7.8-8.5 | 7.8-8.4 | 7.4-8.4 | 7.5-7.8 |
| Dissolved oxygen (mg l^-1^) | 9.2-15.7 | 7.2-15.7 | 1.5-11.7 | 0.3-4.3 |
| Chlorophyll-a (µg l^-1^) | 1.5-27.9 | 1.8-28.5 | 1.8-26.2 | 0.2-27.7 |
| Dissolved organic carbon (mg l^-1^) | 1.9-8.5 | 2.1-5.1 | 2.1-7 | 7-55.1 |
| Prokaryotic abundance (ml^-1^) | 1*10^6^-2.7*10^6^ | 0.9*10^6^-5.8*10^6^ | 2*10^6^-10.8*10^6^ | 5.3*10^6^-24.7*10^6^ |
| Virus like particles (ml^-1^) | 1*10^7^-6*10^7^ | 2.3*10^7^-6.4*10^7^ | 1*10^7^-15.2*10^7^ | 5.7*10^7^-68.3*10^7^ |

Supplementary table 2 ANOSIM comparing the site categories across the sampling period of bacterioplankton community composition (BCC) and extracellular enzymatic activity (EEA) respectively (999 permutations, p values were Bonferroni corrected).

|  |  | BCC |  |  |  | EEA |  |  |
| --- | --- | --- | --- | --- | --- | --- | --- | --- |
| Category |  | Main channel | Dynamic sites | Semi-isolated sites |  | Main channel | Dynamic sites | Semi-isolated sites |
| Dynamic sites | R  *p* | n.s. | - | - |  | n.s. | - | - |
| Semi-isolated sites | R  *p* | 0.40  *0.006* | 0.28  *0.006* | - |  | 0.34  *0.006* | 0.14  *0.03* | - |
| Isolated site | R  *p* | 0.55  *0.006* | 0.80  *0.006* | 0.80  *0.006* |  | 0.64  *0.006* | 0.72  *0.006* | 0.59  *0.006* |

Supplementary table 3 Initial pigment ratio matrix for CHEMTAX analysis. fuco (fucoxanthin), neo (neoxanthin), perid (peridinin), viola (violaxanthin), diadino (diadinoxanthin), allo (alloxanthin), lut (lutein), zea (zeaxanthin), cantha (canthaxanthin), chl-b (chlorophyll-b), echin (echeninone), α-car (α-carotene), chl-a (chlorophyll-a)

| Group\|Pigment | fuco | neo | perid | viola | diadino | allo | lut | zea | cantha | chl-b | echin | α-car | chl-a |
| --- | --- | --- | --- | --- | --- | --- | --- | --- | --- | --- | --- | --- | --- |
| Cyanobacteria | - | - | - | - | - | - | - | 0.252 | 0.026 | - | 0.077 | - | 1 |
| Diatoms | 0.620 | - | - | 0.001 | 0.127 | - | - | 0.004 | - | - | - | - | 1 |
| Chrysophytes | 0.251 | - | - | 0.103 | 0.009 | - | - | 0.011 | - | - | - | - | 1 |
| Chlorophytes | - | 0.036 | - | 0.036 | - | - | 0.268 | 0.017 | - | 0.317 | - | 0.002 | 1 |
| Cryptophytes | - | - | - | - | - | 0.367 | - | - | - | - | - | 0.026 | 1 |
| Dinoflagellates | - | - | 0.493 | - | 0.194 | - | - | - | - | - | - | - | 1 |
| Euglenophytes | - | 0.023 | - | - | 0.239 | - | - | - | 0.003 | 0.241 | 0.009 | - | 1 |

References

Descy JP, Higgins HW, Mackey DJ, et al (2000) Pigment ratios and phytoplankton assessment in northern Wisconsin lakes. J Phycol 36:274–286. doi: 10.1046/j.1529-8817.2000.99063.

Fasching C, Behounek B, Singer GA, Battin TJ (2014) Microbial degradation of terrigenous dissolved organic matter and possible consequences for carbon cycling in brown-water streams. Sci Rep 4:1–3. doi: http://dx.doi.org/10.1038/srep04981

Greisberger S, Teubner K (2007) Does pigment composition reflect phytoplankton community structure in differing temperature and light conditions in a deep alpine lake? An approach using HPLC and delayed fluorescence techniques. J Phycol 43:1108–1119. doi: 10.1111/j.1529-8817.2007.00404.x

Helms, J R, Stubbins A. Ritchie J D, Minor E C, Kieber D J, Mopper K. (2008) Absorption spectral slopes and slope ratios as indicators of molecular weight, source, and photobleaching of chromophoric dissolved organic matter. Limnol.Oceanogr 53: 955–969.

Lauridsen TL, Schluter L, Johansson LS (2011) Determining algal assemblages in oligotrophic lakes and streams: comparing information from newly developed pigment/chlorophyll a ratios with direct microscopy. Freshw Biol 56:1638–1651. doi: 10.1111/j.1365-2427.2011.02588.x

Mackey MD, Mackey DJ, Higgins HW, Wright SW (1996) CHEMTAX - A program for estimating class abundances from chemical markers: Application to HPLC measurements of phytoplankton. Mar Ecol Prog Ser 144:265–283. doi: 10.3354/meps144265

McKnight DM, Boyer EW, Westerhoff PK, et al (2001) Spectrofluorometric characterization of dissolved organic matter for indication of precursor organic material and aromaticity. Limnol Oceanogr 46:38–48

Ohno T (2002) Fluorescence inner-filtering correction for determining the humification index of dissolved organic matter. Environ Sci Technol 36: 742–746.

Parlanti E, Wo K, Geo L, Lamotte M (2000) Dissolved organic matter ¯ uorescence spectroscopy as a tool to estimate biological activity in a coastal zone submitted to anthropogenic inputs. 31:1765–1781

Schagerl M, Donabaum K (2003) Patterns of major photosynthetic pigments in freshwater algae. 1. Cyanoprokaryota, Rhodophyta and Cryptophyta. Ann Limnol J Limnol 39:35–47. doi: 10.1051/limn/2003003

Schluter L, Lauridsen TL, Krogh G, Jorgensen T (2006) Identification and quantification of phytoplankton groups in lakes using new pigment ratios - a comparison between pigment analysis by HPLC and microscopy. Freshw Biol 51:1474–1485. doi: 10.1111/j.1365-2427.2006.01582.xß

Stedmon CA, Markager S, Bro R (2003) Tracing dissolved organic matter in aquatic environments using a new approach to fluorescence spectroscopy. Mar Chem 82:239–254. doi: 10.1016/S0304-4203(03)00072-0

J.L. Weishaar J L, Aiken G R, Bergamaschi B A, Fram M S, Fujii R, Mopper K (2003) Evaluation of specific ultraviolet absorbance as an indicator of the chemical composition and reactivity of dissolved organic carbon. Environ Sci Technol 37: 4702-4708

Wright SW, Jeffrey SW, Mantoura RFC, et al (1991) Improved HPLC method for the analysis of chlorophylls and carotenoids from marine phytoplankon. Mar Ecol Prog Ser 77:183–196. doi: 10.3354/meps077183
